# Supplementary material for: Commensal Bacterium Rothia aeria Degrades and Detoxifies Gluten via a Highly Effective Subtilisin Enzyme
Source: Nutrients. 2020 Dec 2;12(12):3724. doi: 10.3390/nu12123724 (PMC7761627; doi:10.3390/nu12123724)
Supplement: Supplementary file 1 [file nutrients-12-03724-s001.pdf]

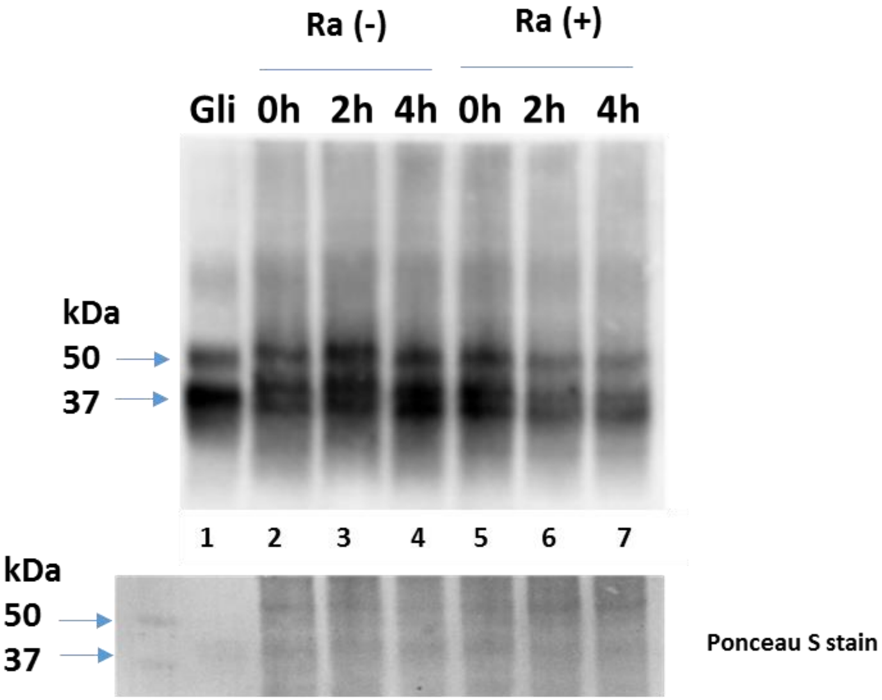

Supplemental Figure 1 Enzymatic degradation of gliadins in mouse chow *in vitro* detected by immunoblotting. Mouse chow was supplemented with *R. aeria* (Ra+) and without (Ra-) and incubated at 37ºC for 0, 2 and 4 hr. Aliquots (10 µl) of 60% ethanol-extracted sample supernatants were loaded. Lane1: Gliadin control (Gli, 15 µg); Lane 2: Ra (-) at 0 hour; Lane 3: Ra (-) at 2 hr; Lane 4: Ra (-) at 4 hr; Lane 5: Ra (+) at 0 hr; Lane 6: Ra (+) at 2 hr; Lane 7: Ra (+) at 4 hr, blue (arrow) points to the gliadin bands at 37 and 50 kDa.

MIC Susceptibility of Two Rothia Species to Antimicrobics

Category		Antimicrobics	MIC (µg/ml)	
			Rm	Ra
Aminoglyosides		Amikacin	<4	<4
		Gentamicin	<1	<1
		Streptomycin	<1000	<1000
		Tobramycin	4	8
Beta-lactams				
	Cephalosporins	Cefepime	<2	<2
		Cefotaxime	<1	<1
		Ceftazidime	4	8
		Ceftaroline	<0.12	<0.12
		Ceftriaxone	<8	<8
	Carbapenems	Doripenem	<0.12	0.25
		Ertapenem	<0.25	0.5
		Imipenem	<1	<1
		Meropenem	<1	<1
	Monobactams	Aztreonam	>16	>16
	Penicillins	Ampicillin	<0.12	<0.12
		Oxacillin+2%NaCl	<0.25	<0.25
		Penicillin	<0.06	<0.06
Glycopeptides		Vancomycin	<1~1	2
		Telavancin	<0.06	0.12
Glycylcyclines		Tigecycline	<0.06	0.12
Lincosamides		Clindamycin	<0.5~0.5	>2
Lipopeptides		Daptomycin	0.5~1	4
Macrolides		Erythromycin	<0.25	4
Nitrpfurans		Nitrofurantoin	<32	<32
Oxazolidinones		Linezolid	<1~1	1
Polymyxins		Colistin	>4	>4
		Polymixin B	>4	>4
Quinolones		Ciprofloxacin	2~4	2~4
		Gatifloxacin	<1	<1
		Levofloxacin	<1~2	1
		Moxifloxacin	<0.25	0.5
Rifamycins		Rifampin	<0.5	<0.5
Tetracyclins		Doxycycline	<2	<2
		Minocycline	<2	<2
		Tetracycline	<2	<2
Others		Chloramphenicol	<2	<2
Combinations		Piperacillin / tazobactam constant 4	<8/4	<8/4
		Quinupristin / dalfopristin	0.25	0.12
		Ticarcillin / clavulanic acid constant 2	<16/2	<16/2
		Trimethoprim / sulfamethoxazole	<0.5/9.5	<0.5/9.5

Supplemental Figure 2, MIC (minimal inhibitory concentration) susceptibility of two *Rothia* species to antibiotics The experiment was performed by using MIC susceptibility plates (Sensititre, Thermofisher Scientific/ Trek diagnostic systems). MIC was recorded as the lowest concentration of antibiotic that inhibits visible growth . Rm: *Rothia musinoginosa*; Ra: *Rothia aeria*

There are some concerns that *Rothia* bacteria may cause side effects in patients if they colonize non-naturally areas of the human body. Here data shows that Rm and Ra are susceptible to antibiotics and to 70% ethanol (Figure 2 A). Notably, the dead Rm/Ra still exhibit gluten degrading activity (Figure 2 B-D), indicating that these inactivated Rm and Ra maintain gluten proteolytic activity to benefit CD patients.

>BAV86562.1 glycerol-3-phosphate ABC transporter [*Rothia aeria*]

MAITAGLPATAAPAGDPDTPVAQDIARNSREHAVLSDSMKKAEGNIPVFVQFKGKGAYEQTQSPAVLANKQAPTNKQ
AEVQAIKTQVQSQAQAAAQSTGAKTLYTTHNIMRGVALQGDAAQIRALANNPEVERITPIVPKKKQNAGSVVDTGAA
ENWARENSGYT**GKDVKIAVVDSGIDYTHADFGGPGTVEAFNKATKLTEMPAADSGLYDAKKYIGGYDLVGDSYDGTN**
QTAPDNNPIDCSAGGHGTHVAGTAAGYGVNQDGTTFRGDYSKLTAEQLNQMKIGPGAAPEAQLYSFRVFGCTGTTGV
VVQALDRTLDPNGDGDFSDRANIVNLSIGGEFSPPDDADAYAVESLNRQGVLAVVSAGNATDYYGRGDTYSDSGQPA
NAVSALTVANSIGSSYAVDSMEIQAPANVAGKVPGDYTVSYTYTGAKPEALTGTVVTPSESNKFGCEAFSAEDAAKI
KDKWVFLEWANADGSLPCGSKVRFDNVEKAGGKGVVLSSEEEKPALPIGGNESIPGFRVAKSASAKVREAAANGELK
VRLGTDLKESLRVPSNKKDQLTASSARGYHGTYGYTKPDVAAPGNNISSARVGTGTDGISYTGTSMSAPFAAGVAAQ
VLQANQSYGPTQLKAAIMNSANHDVRTADGNVYAVDRVGSGRIDAKAAAETKVLLYNADRPAQVSQTFGVLEYAVNE
GKQTLTREMTVENFDSHTHTYNISYAGSTDMPGVEFSLPSNITVNPGEKKNFTVTITIDPAAMEKTMDPAMEKTHNS
VDPYGDGTELVPEQYRQFIASESGRILLTEGAATLRAPIHAAPKPASAMKVEGSSVEIPAGEHQANLKLTGTELNQR
GYKSLLGAFEHGASIERTSPVKLDVSSNAKANMQHVGAASTAPALKASGGNPNDGLLAFGISTWANWDVVSTENTFT
VNIDTDGNNRADYMLVTDRAKGIDFPIVRLYGYKNGNLEQIAYYPLNNAWGDTDTNMMDSNALVMAVPLKDLGLSAE
KTKDIKYSVSATTQYAWTNVSETGWINYRPFDPKLWFSGTAATVPGFFADAPSSELVAHRAEGATDVKALFLHMHNT
TGDLSGLNGAAGNRAQVLEVTEQQQLDPAPSRFTDVPAENQFYAEINWLAQRRITTGYPDGTFRPGENVERGAMAAY
FYRLAGTPQFTAPDNPTFSDVPKSHPFYKEIEWMAARGITTGYGDGTFRPSASVNRDAMAAFFYRYANSPQFAAPAA
SPFKDVPANSQFYKEIAWLAEQGITKGWDDGTYRPGEPIHRDAMAAFLYRYSDKVLK

Supplemental Figure 3. Amino acid sequence of *R. aeria* BAV86562.1. In bold: domain cd07474 representing the peptidase S8 family domain in Vpr-like proteins. BAV86562.1 is not an ABC transporter protein as its name suggests, since it lacks the LSGGQ domains characteristic of the ABC transporter family. The highlighted D, S and H residues comprise the catalytic triad of the subtilisin enzyme.
